# Regulation of DNA Double-Strand Break Repair by Non-Coding RNAs

**DOI:** 10.3390/molecules23112789

**Published:** 2018-10-27

**Authors:** Roopa Thapar

**Affiliations:** Department of Molecular and Cellular Oncology, University of Texas M.D. Anderson Cancer Center, Houston, TX 77030, USA; rthapar@mdanderson.edu; Tel.: +1-713-201-7875

**Keywords:** DNA repair, long non-coding RNA, microRNA, DNA damage, double-strand breaks, NHEJ, HR

## Abstract

DNA double-strand breaks (DSBs) are deleterious lesions that are generated in response to ionizing radiation or replication fork collapse that can lead to genomic instability and cancer. Eukaryotes have evolved two major pathways, namely homologous recombination (HR) and non-homologous end joining (NHEJ) to repair DSBs. Whereas the roles of protein-DNA interactions in HR and NHEJ have been fairly well defined, the functions of small and long non-coding RNAs and RNA-DNA hybrids in the DNA damage response is just beginning to be elucidated. This review summarizes recent discoveries on the identification of non-coding RNAs and RNA-mediated regulation of DSB repair.

## 1. Introduction

One of the most remarkable advances in molecular biology during the last decade is the annotation of the transcriptome in numerous organisms and the discovery that ~75% of the genome is transcribed into noncoding RNAs, but only ~2% of the transcriptome accounts for proteins [1,2,3]. These noncoding RNAs are involved in a diverse array of biological functions that are only beginning to be understood. A strong connection has been established between several small and long noncoding RNAs (lncRNAs) and human disease, especially cancer. MicroRNA signatures are linked to cancer metastasis and tumor progression. Several microRNAs and lncRNAs are oncogenes or tumor suppressors that regulate the DNA damage response and genomic stability. Understanding the mechanisms by which they act opens up new opportunities for intervention in cancer.

Double-strand DNA breaks (DSBs) are the most lethal form of DNA damage in cells and are predominantly repaired either by Non-Homologous DNA End Joining (NHEJ) [4,5,6] or Homologous Recombination (HR) pathways [7,8]. DSBs can occur endogenously as a result of unrepaired mutations that lead to stalled replication forks, due to inhibition of DNA replication culminating in breaks during mitosis, or because of the harmful effects of ionizing radiation [9,10,11]. Large-scale genome rearrangements such as deletions, insertions, and translocations that are highly genotoxic, are a consequence of defects in DSB repair and are positively correlated with cancer progression [12]. Germ line mutations in proteins involved in HR can cause several diseases such as Fanconi Anemia (FA) [13,14], breast and ovarian cancers [15,16], Bloom syndrome [17,18], Werner syndrome [19], and ataxia telangiectasiam Nijmegen breakage syndrome [20,21], whereas defects in NHEJ can result in chromosomal instability, promoting tumorigenesis, radiosensitive severe combined immunodeficiency (RS-SCID) [22,23], microcephaly, and growth defects in children [24,25,26]. A third mechanism of repair called microhomology-mediated end joining (MMEJ) or Alt-NHEJ is a minor pathway of DSB repair, and is triggered in the event that HR is not capable of repairing breaks [27,28,29,30]. Pathway choice for double strand break repair is complex and is dependent on the type of DSB induced, the cell cycle, and the activity of the repair components [31,32,33]. Understanding the molecular mechanisms involved in repair of DSBs has broad implications for a range of human diseases such as cancer, as well as aging [8,34].

In mammals, NHEJ is the primary DSB repair pathway that is active throughout the cell cycle, although it is more prominent during the G0 and G1 phases of the cell cycle [35,36,37,38]. NHEJ is used to repair double-strand DNA breaks (DSBs) that arise due to V (D) J recombination [39,40], ionizing radiation [41], and reactive oxygen species [42], without the need for a template DNA. The first step in NHEJ involves binding of Ku70–Ku80 heterodimers to both ends of a DNA break [43,44,45], followed by the recruitment and activation of the catalytic subunits of DNA dependent protein kinase (DNA-PKcs) [46,47]. DNA end processing involves phosphorylation of nucleases such as Artemis, and DNA polymerases to replace damaged bases; followed by ligation of blunt DNA ends by the XLF-XRCC4-DNA ligase IV complex. Recently, a triple-strand break repair model has been proposed in which ribonucleotide incorporation at the break termini is important for the ligation step of NHEJ [48]. Due to direct joining of the DNA ends that frequently involves DNA end resection, NHEJ is an error-prone DNA repair mechanism. 

Unlike NHEJ, HR occurs only in rapidly dividing cells during the S and G2/M phases of the cell cycle and requires a sister chromatid as a template for repair [35,49,50]. The early events in HR can be characterized as (1) signaling to the DSB (2) relaxing chromatin to open the site of the break for repair, and (3) resection of the 5’ end of the DNA end [51,52,53]. The kinetics of the reaction measured in vivo, based on fluorescence recovery after photobleaching (FRAP) experiments after UV-based laser irradiation experiments, is rapid [54]. Within ~1 sec of the damage, the DSB is recognized by poly (ADP-ribose) polymerase 1 (PARP1), followed by recruitment of the chromatin remodeler Alc1 to the PAR chains, relaxation of chromatin allowing access of the helicase-nuclease “sensor” Mre11-Rad50-Nbs1 (MRN) complex to the site of damage [55,56,57]. Recruitment of MRN to the DSB site has been reported to occur within ~13 s and appears to be via direct association with PAR chains. Single-molecule localization experiments show that the temporal dynamics and spatial distribution of MRN foci are cell type-specific, and the MRN complex is recruited after *γ*H2AX foci have already formed [58,59]. In addition to MRN, the phosphatidylinositol 3-kinase-like protein kinase (PIKK) ATM is activated by autophosphorylation [60]. ATM phosphorylates the DNA repair factors BRCA1, CtIP, EXO1 that act downstream in the reaction mechanism to promote resection, as well as the histone variant H2AX, which is phosphorylated at Ser139 to generate *γ*H2AX [50]. Deposition of *γ*H2AX occurs at the site of the DSB and is amplified, spreading for hundreds of kilobases from the damage site. These initial factors recruited to the sites of DNA damage form distinct DNA repair foci, the nature of which remains ambiguous. The amplification of this damage is dependent on formation of the *γ*H2AX-MDC1 complex that recruits the ubiquitin E3 ligase RNF8 [61]. RNF8-mediated ubiquitylation of histones H2A and H2AX results in decondensation of chromatin and recruitment of RAP80, ABRA1, and BRCA1 [61]. RNF8 also interacts with other chromatin remodeling complexes such as CHD4, a component of the nucleosome remodeling and deacetylase complex, NuRD [62]. After repair has occurred, chromatin is remodeled to its initial state. Resection of the 5’ ends of the DSB is promoted by MRN, BRCA1, CtIP, and Exo1 nuclease and helicase activities, leaving ssDNA, which is rapidly bound by replication protein A (RPA) [50,63]. RPA is then exchanged for Rad51 in the presence of BRCA2 [50]. The later steps in HR involve strand invasion to the homologous template followed by DNA synthesis. This can occur by either the double-strand break repair pathway (DSBR) that results in chromosomal crossover, or the synthesis-dependent strand-annealing (SDSA) pathway in which there is strand displacement after DNA synthesis. Precise assembly of a multitude of protein factors and macromolecular machines is required for repair to occur. Regulators such as kinases, ubiquitin ligases, and acetyl-transferases that remodel chromatin are also essential to temporally and spatially control DSB repair. 

HR and NHEJ DNA repair mechanisms are conserved in evolution and occur in both eukaryotes and prokaryotes. There are several excellent reviews on the detailed mechanisms and protein factors involved in DSB repair [64,65,66,67,68,69]. Recent studies indicate that non-coding RNAs (ncRNAs), that include long ncRNAs (lncRNAs) as well as small ~21 nt RNAs such as microRNAs (miRNAs), DNA damage induced RNAs (diRNAs), and Drosha- and Dicer-dependent small RNAs (DDRNAs) play important roles in the DNA damage response [70,71,72,73]. This review focuses on emerging themes in the roles of ncRNAs in DSB repair that are observed in humans and have direct cancer relevance.

## 2. LncRNAs

Long non-coding RNAs are > 200 nt RNA Pol-II transcripts that, in general, are not translated into proteins [74,75]. It is projected that >30,000 lncRNAs are expressed in humans, greater than the number of protein-coding genes (~20,000) in the genome [76,77,78]. These transcripts are polyadenylated, capped at the 5’ end, and spliced into mature RNAs, exported into the cytoplasm and are regulated like protein-coding mRNAs, although the mechanisms of RNA processing might differ from mRNAs [79]. Based on their proximity to protein coding genes, they have been classified as sense, antisense, pseudogenes, intronic, and intergenic (also called lincRNAs) [80,81]. Although the functions of most lncRNAs are poorly understood, in several cases such as the Xist RNA [82,83] that is required for mammalian dosage compensation and X-chromosome inactivation, they are not artifacts of pervasive transcription of “junk DNA”, and play important biological roles in control of the cell cycle, in development, and have been linked to cancer progression [84,85]. 

The mechanisms by which lncRNAs act are varied. Some lncRNAs exert epigenetic functions by acting as scaffolds for chromatin-modifying complexes, e.g., *HOTAIR* is associated with chromatin and recruits the polycomb repressive complex 2 (PRC2) and the LSD1/CoREST/REST demethylase complexes to specific loci in the genome for gene silencing [86,87]. Chromatin-associated lncRNAs such as promotor RNAs (pRNAs) are tethered to specific sites in the genome by forming RNA-DNA hybrids thereby acting in *cis,* where they recruit PARP1, the ATP-remodeling complex NoRC, and the methyltransferase Dnmt3b to silence rDNA [88,89]. LncRNAs can also directly regulate transcription by acting as transcription coregulators as in the case of the steroid receptor RNA activator *SRA* that activates transcription with nuclear receptors [90,91]. An example of a corepressor is the ncRNAs transcribed from the *CCND1* gene (cyclin D1) locus that can bind the TLS protein that in turn turns-off the histone acetyl transferase activity of p300/CBP [92]. Another role of lncRNAs is in formation of nuclear bodies or foci. Paraspeckles are nuclear bodies that trap adenosine-to-inosine edited RNAs and paraskeckle formation relies on the lncRNA *NEAT1* [93,94]. Similarly, *MALAT1* (or *NEAT2*) is a component of nuclear speckles where it retains serine/arginine (SR) splicing factors and affects splicing [95,96]. Besides their roles in the nucleus, several antisense lncRNAs can hybridize with the 3’ untranslated regions (3’ UTRs) of mRNAs to regulate their stability in the cytoplasm and/or interact with RNA decay factors and miRNAs. Examples of antisense transcripts are lncRNAs produced from the 3’ UTR of *iNOS* [97] and half-STAU1-binding site RNAs (1/2sbsRNAs) [98]. The lncRNAs *PTENP1* and *KRASP1* [99] sequester miRNAs and act as miRNA sponges thereby affecting RNA levels of a range of mRNAs in the cell.

## 3. LncRNAs and the DNA Damage Response

Given the large number of lncRNAs and their diverse functions, it is not surprising that several lncRNAs are induced upon DNA damage and have been proposed to play a role in double-strand break repair (Table 1). Oncogenes as well as tumor suppressor lncRNAs have been documented. The first lncRNAs (ncRNA*_CCND1_*) identified as being transcribed in response to DNA damage signals (ionizing radiation), were from the *CCND1* gene (cyclin D1) locus [92,100]. The ncRNA*_CCND1_* (between 200–330 nts and greater) exist on chromatin as RNA: DNA hybrids as well as ssRNA. 

RNA immunoprecipitation and ChIP experiments established TLS (translocated in liposarcoma) as the protein that was recruited to ncRNA*_CCND1_*. RNA bound TLS results in an allosteric change that allows TLS to bind and inhibit the histone acetyltransferase complex CBP/p300, resulting in transcription repression. Since then, several damage-induced lncRNAs have been identified.

The mechanisms by which lncRNAs modulate DSB repair include: (i) modulating the activity of p53 either at the level of transcription, translation or posttranslational modifications; (ii) by acting as tethers for recruitment of chromatin remodeling complexes to the site of damage; (iii) by sequestering negative regulators of DNA repair away from the damage site as decoys; (iv) by acting as scaffolds that directly interact with several DNA repair proteins such as Ku70/Ku80, BRCA1, 53BP1, Mre11, PARP1; (v) by sequestering miRNAs that regulate the stability of DNA repair proteins, thereby modulating mRNA expression levels. As noted below and in Table 1, a subset of lncRNAs are part of a p53 network i.e., either under direct p53 control or they modulate the activity or expression of p53. In this section, I highlight some of the unique lncRNAs that have clear cancer relevance. A summary of the roles of ­DNA damage induced lncRNAs in specific disease states and development is given in Table 2 (at the end of Section 3). 

### 3.1. p53 Linked LncRNAs

The transcription factor p53 plays key roles in normal cell proliferation and tumor suppression and is mutated in over 50% of human cancers. In response to DNA damage, it is involved in transactivation or repression of target genes to induce either cell cycle arrest or apoptosis. Not surprisingly, several lncRNAs have been discovered that are either transcription targets of p53 or regulate the p53-dependent gene expression signature [129,130]. Notably, the intergenic lncRNA that resides ~15 Kb upstream of the p53 target *CDKN1A* (or p21), called lincRNA-p21 [101,104,131] was identified as a repressor of the p53 transcriptional response, decreasing gene expression of hundreds of p53 target genes and triggering apoptosis. lincRNA-p21 is a large ~3.1 Kb transcript that is a distinct gene with its own promoter and is transcribed in the opposite orientation to *CDKN1A* in response to DNA damage. LincRNA-p21 likely functions through its interaction with heterogeneous nuclear ribonucleoprotein-K (hnRNP-K), which is a component of repressor complexes, and is recruited to promoters of p53 target genes. In addition to its roles in the nucleus, lincRNA-p21 is also reported to regulate translation in the cytoplasm [132]. A similar mechanism is employed by the DNA damage induced lncRNA called *PANDA* (for p21 associated ncRNA DNA damage activated) which is also induced by p53 and is transcribed antisense ~5 Kb upstream of *CDKN1A* [104]. *PANDA* acts as a decoy for the nuclear transcription factor Y subunit alpha (NF-YA) to repress the pro-apoptotic genes *FAS* and *BIK*. The *APELA* lncRNA is a positive regulator of p53 [104,133]. It binds to heterogeneous nuclear ribonucleoprotein-L (hnRNP-L) to inhibit the hnRNP-L-p53 interaction, thereby promoting p53-induced apoptosis. A more recent study [105] identified the lncRNA *DINO* (Damage Induced Noncoding), a ~951 bp RNA that is induced by p53 and is also located upstream of *CDKN1A.* Intriguingly, *DINO* interacts directly with p53 and stabilizes it, promoting the transactivation functions of p53. *DINO* knockout mice are also deficient in p53-dependent gene expression in response to DNA damage. Several lncRNAs have been shown to bind the repressor PRC2 that has H3K27 trimethylase activity. *PINT* is a lincRNA that is transcriptionally activated by p53 and is a positive regulator of cell proliferation and cell survival in mouse cells and a negative regulator in human cells. In both mice and humans, *PINT* targets PRC2 to specific gene loci for repression, thereby affecting cell proliferation, although the outcomes appear to be different in mice vs. humans [102,103]. *TUG1* is a lincRNA transcript that is upregulated by p53 and may act on chromatin to downregulate p53 mediated transcriptional pathways [112,113]. *TUG1* interacts with PRC2 and additional corepressor complexes that include histone methyltransferases, demethylases, and chromatin modifiers [112,113]. 

#### 3.1.1. WRAP53 (WD Repeat Containing Antisense to p53)

**Figure 1:**
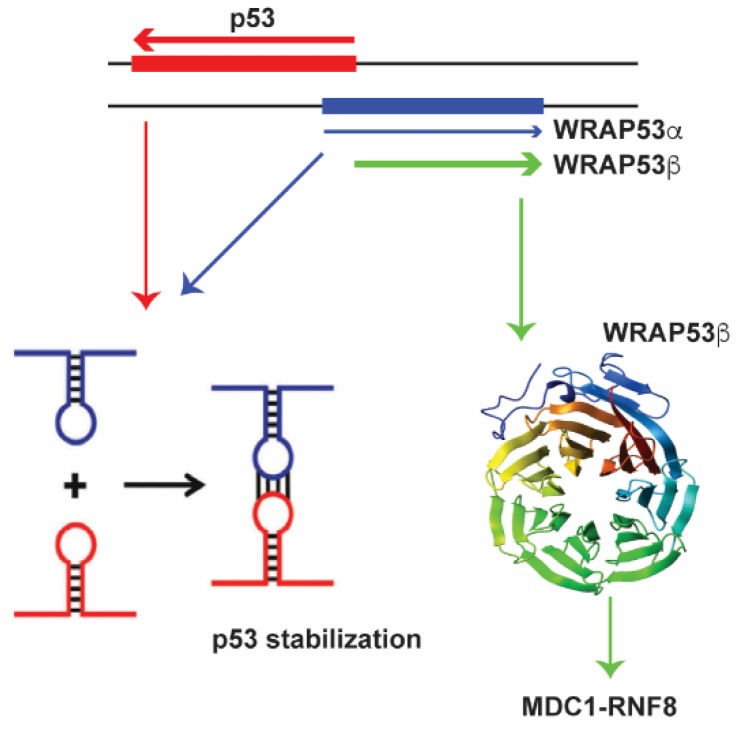
The antisense lncRNA *WRAP53α* (shown in blue) directly interacts with the p53 mRNA (shown in red) to stabilize it, affecting p53 protein levels. The WRAP53*β* (in green) recruits and stabilizes RNF8 at double strand breaks.

A fascinating example of a lncRNA that is partially antisense to *p53* and induces p53 levels upon DNA damage is *WRAP53* [134,135] (Figure 1). 

Antisense lncRNA transcripts of *p53* that overlap with the first *p53* exon are called *WRAP53α* [135] and those that overlap with the first intron are called *WRAP53γ*. The *WRAP53α* RNA directly interacts with the p53 mRNA via RNA-RNA interactions to affect p53 protein levels [135]. WRAP53 an interesting example of gene that encodes both a lncRNA and is also transcribed into the protein WRAP53*β* (also known as WDR79 or TCAB1) [136,137,138] WRAP53*β* has multiple functions in the cell one of which is to target RNF8 to DNA double-strand breaks. Therefore both the protein, as well as the lncRNA is involved in the DNA damage response, albeit through different mechanisms.

#### 3.1.2. LINP1

**Figure 2:**
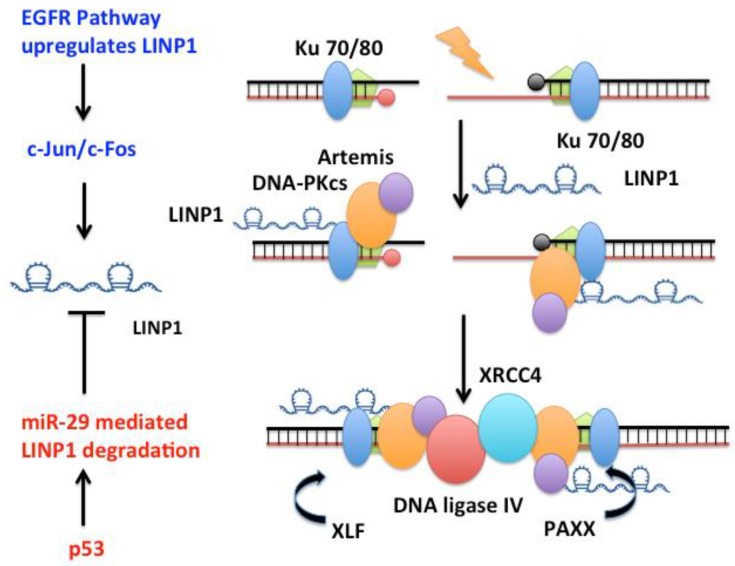
*LINP1* is a 917 nt long RNA that promotes the assembly of NHEJ factors to enhance DNA repair in Triple Negative Breast Cancers (TNBC). LINP1 directly associates with Ku70/Ku80 as well as DNA-PKcs to increase NHEJ activity in TNBC tumors. Activation of EGFR signaling via MEK and JNK kinases up regulates LINP1 whereas the tumor suppressor TP53 represses LINP1 via the microRNA miR-29.

The long non-coding RNA “lncRNA in non-homologous end joining pathway” LINP1 [106,139,140] forms an RNA scaffold that, at least in cell extracts, interacts with both Ku70–Ku80 and DNA-PKcs on chromatin to promote end joining (Figure 2). LINP1 is overexpressed in triple negative breast cancer (TNBC) tumors, in the TNBC cell lines MDA-MB-231 and MDA-MB-468, and in triple-negative immortalized mammary cells MCF10A. LINP1 is not detected in ER-positive MCF7 cells. NHEJ reporter assays show that knockdown of LINP1 in TNBC cells decreases NHEJ and overexpression of LINP1 in MCF7 cells increases NHEJ activity [106]. Knockdown of LINP1 also sensitizes mice to ionizing radiation due to defects in DNA repair via the NHEJ pathway. Activation of EGFR signaling via MEK and JNK kinases up regulates LINP1 whereas tumor suppressor p53 represses LINP1 via the microRNA miR-29. Recent studies show that LINP1 levels are also increased in cervical cancer [141] as well as advanced prostate cancer [139] and are correlated with poor prognosis. 

#### 3.1.3. MALAT1 or NEAT2

**Figure 3:**
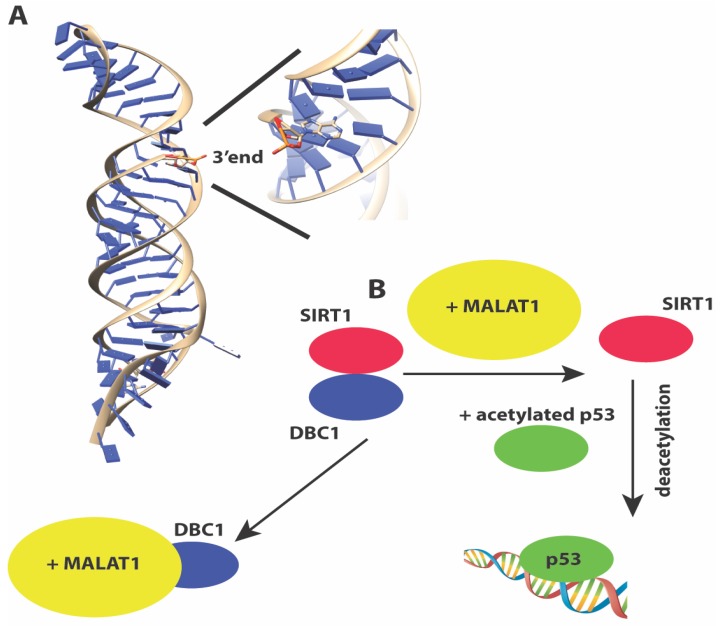
(**A**) Structure of the 3’ end of *MALAT1* reveals a triple helix with the 3’ end sequestered in a U-A-U base triple, protecting the 3’ end from nucleolytic degradation (**B**) *MALAT1* sequesters the negative regulator DBC1, releasing the deacetylase SIRT1 to activate p53 for transactivation.

Metastasis-associated lung adenocarcinoma transcript 1 or *MALAT1* [110,111] is a ~7 Kb lincRNA that is evolutionarily conserved and is associated with many cancers [142]. It was originally identified in a screen for lncRNAs in early-stage non-small cell lung cancers that were metastatic. *MALAT1* performs a wide array of functions. It co-localizes with nuclear speckles [143] and is important for pre-mRNA processing and splicing [96,144,145]. The longer *MALAT1* transcript can be further processed into a short 61 nt tRNA-like fragment called *MALAT1*-associated small cytoplasmic RNA (mascRNA) that regulates translation in the cytoplasm [146]. *MALAT1* is one of the few lncRNAs for which structural information is available for at least a portion of the RNA. The 3’ end of *MALAT1* forms an expression and nuclear retention element (ENE)+A, a triple helix element for which a 3.1 Å crystal structure is available (Figure 3A). The structure shows how the triple helix sequesters the 3’ end of *MALAT1* in a U-A-U base triple, and protects the 3’ end of the lncRNA from decay [147]. 

Like *TUG1, MALAT1* is also upregulated by p53 upon DNA damage. A proteomics screen has identified numerous proteins involved in transcription, RNA processing, translation, protein degradation, and metabolism as *MALAT1* interacting proteins [148]. *MALAT1* indirectly regulates p53 protein transactivation function by sequestering DBC1, a partner for the deacetylase SIRT1, which targets p53 for deacetylation (Figure 3B). Therefore similar to several lncRNAs discussed here, p53 creates a feedback loop to regulate the expression of several lncRNAs, that in turn control p53 function. Besides regulating p53 activity, *MALAT1* has also been reported to exist in a complex with PARP1 and Lig3 in vivo, and may regulate the Alt-NHEJ pathway of double-strand break repair [149]. 

### 3.2. p53-independent lncRNAs

Several lncRNAs (Table 1) are transcribed independent of p53, and act indirectly in DDR by altering signaling pathways or via epigenetic mechanisms that repress transcription of DNA repair genes. The lncRNAs *ANRIL* (antisense noncoding RNA in the INK4 locus), *MDC1-AS* and *lncRNA*-*JADE* are induced by ATM activation. *ANRIL* is encoded from the INK4B-ARF-INK4A locus on chromosome 9p21 and this locus is the most frequent copy number alteration across tumors. *ANRIL* recruits the PRC complex to repress transcription of tumor suppressors INK4B, ARF, INK4A at this locus and indirectly affects HR by altering cell cycle checkpoints. 

The *CUPID1* and *CUPID2* lncRNAs are transcribed in hormone-positive breast cancers and regulate pathway choice in vivo, switching repair from the error-prone NHEJ to HR. However the exact mechanism is unclear. The *TODRA* RNA (transcribed in opposite direction of RAD51) increases the expression of *RAD51* in an E2F1-dependent manner and hence facilitates HR. 

#### 3.2.1. DNA Damage-Sensitive RNA1 (*DDSR1*)

The lincRNA *DDSR1* was identified in microarray screens of RNA isolated from hTert-immortalized human skin fibroblasts treated with the DNA damaging agents neocarzinostatin, campothecin, or etoposide [115]. *DDSR1* was identified as a 1616 nt inter-genic transcript that was upregulated by ~2.5 fold in all samples treated with the DNA damaging drugs. *DDSR1* expression is induced by ATM and under control of NF-*κ*B, however p53 is not required for expression. Although *DDSR1* expression is not under p53 control, *DDSR1* was found to regulate the expression of p53 target genes and *DDSR1* knockdown significantly upregulated p53 targeted mRNAs, particularly those involved in cell proliferation and cell survival. Intriguingly, *DDSR1* knockdown impaired HR by ~50% and *DDSR1* knockdown cells were also more sensitive to the PARP1 inhibitor Olaparib. RNA pull-down experiments identified heterogeneous nuclear ribonucleoprotein U-like 1 (hnRNPUL1) as an RNA binding protein that specifically associated with *DDSR1*, is recruited to sites of double-strand breaks and is important for end resection during HR [150]. In vivo imaging experiments show that depletion of *DDSR1* or hnRNPUL1 leads to an increase in BRCA1-RAP80 at DSBs. This leads to a model in which the *DDSR1*-hnRNPUL1 complex regulates recruitment of BRCA1-RAP80 to sites of DNA breaks. 

#### 3.2.2. Prostate Cancer Associated Transcript 1 (*PCAT-1)*

PCAT-1 is the first lincRNA identified that participates in DSB repair in prostate cancer [118], and extends the “BRCA-ness” paradigm to include lncRNAs, in addition to mutations in DNA repair genes. Increased PCAT-1 levels downregulate BRCA2 and impairs HR, resulting in an increase in *γ*-H2AX foci formation [116]. PCAT-1 is being used as a biomarker for a predictive response to treatment with PARP1 inhibitors due to synthetic lethality. PCAT-1 levels are inversely correlated with RAD51 foci formation when prostate cancer cells are treated with the PARP1 inhibitors olaparib or ABT-888. The mechanism by which PCAT-1 down regulates BRCA2 is unique compared to other lincRNAs that act predominantly via epigenetic pathways. PCAT-1 is a predominantly cytoplasmic lincRNA and the first 250 nt from the 5’ end of the PCAT-1 RNA were sufficient to downregulate the 3’ untranslated region of BRCA2 mRNA via posttranscriptional mechanisms, likely at the level of RNA stability, although the exact mechanism is unknown. PCAT-1 can also regulate c-Myc levels via a microRNA sequestering mechanism [117]. It will be interesting to see whether a similar mechanism of regulation applies to BRCA2 mRNA. 

#### 3.2.3. Telomeric Repeat-Containing RNAs (TERRA)

Telomeres are structures assembled at the ends of chromosomes that protect the chromosome ends from being recognized as double-strand breaks and hence inhibit activation of the DNA damage response [174]. Telomeres have a unique “end problem” in that activation of DDR could either result in end degradation or trigger incorrect recombination events. Telomere ends also need to be replicated, and progressive shortening of telomeres is correlated with aging. TERRA RNAs are unique class of lncRNAs that are transcribed from the ends of chromosomes and consist of the G-rich telomeric repeats (TTAGGG)n in mammalian cells. TERRA RNAs form stacked G-quadruplex structures [175] that can interact with a number of proteins that are part of the shelterin complex for telomere capping, as well as DNA repair factors [123,176,177]. TERRA lncRNAs regulate the activity of telomerase, form heterochromatin at chromosome ends, and are also involved in capping of chromosome ends to protect telomere genomic integrity. During telomere replication, the ssDNA is bound by RPA, which triggers the ATR checkpoint for repair. The shelterin component POT1 is believed to compete with RPA and inhibit the ATR mediated checkpoint [178]. TERRA RNAs play a role in switching RPA for POT1. In addition, upon depletion of the shelterin protein, telomeric repeat factor 2, TERRA RNA levels increase and TERRA interacts with the hisone demethylase LSD1 [122]. This interaction stimulates the exonuclease activity of Mre11 to trim the 3’ G overhangs of uncapped telomeres and form heterochromatin at telomeric ends [122,177]. Although TERRA RNAs are a very specialized lncRNA family, structure/function studies of TERRA interactions with chromatin modifiers and DNA repair proteins provides important insights into lncRNA function in DSB repair. 

## 4. Small ncRNAs

In mammals, two classes of small non-coding RNAs have been identified as major players in the DNA damage response. The first class consists of microRNAs (miRNAs) which are small, phylogenetically conserved ~22 nt RNAs that negatively regulate the translation and stability of mRNAs via their association with Argonaute (Ago) proteins. miRNAs associate with 3’ untranslated regions (3’UTRs) of mRNAs via RNA-RNA interactions to trigger translational repression or mRNA decay. The second class consisting of ~21 nt small RNAs has recently been identified in response to DNA damage, and are produced from sequences around the DSB. These ncRNAs are called DSB-induced small RNAs or diRNAs in plants and Drosha- and Dicer-dependent small RNAs (DDRNAs) in mammals. Although the same enzymes that are involved in the miRNA pathway generate DDRNAs and diRNAs, their biogenesis and mechanism is distinct from miRNAs, and is discussed in greater detail below. 

### 4.1. miRNAs Involved in the DNA Damage Response

**Figure 4:**
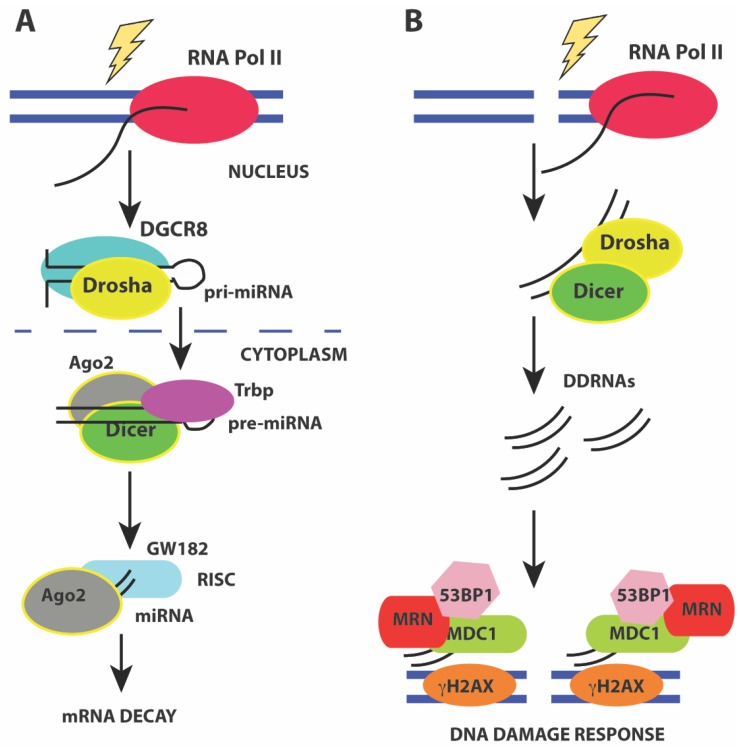
(**A**) pathway of miRNA processing to regulate mRNA decay. Pri-miRNA precursors are transcribed and processed before assembling into a macromolecular complex containing the Drosha/DGCR8 heterodimer to release a ~70 nt pre-miRNA hairpin in the nucleus. The pre-miRNA hairpin is exported to the cytoplasm by XPO5 and Ran-GTP. In the cytoplasm, the pre-miRNA hairpin is cleaved by Dicer to generate the mature ~22 nt miRNA duplex that associates with Ago2 leading to the formation of the RNA-induced silencing complex (RISC). Activation of mRNA decay results in base-pairing of the guide strand to the mRNA 3’ UTR and recruitment of mRNA decay factors such as GW182; (**B**) Activation of the DNA damage response by DDRNAs also involves miRNA processing factors Drosha and Dicer that are required to generate small ~20–35 nt RNAs from the sequences around the site of damage. Formation of DNA damage foci and recruitment of DNA damage factors requires DDRNAs, Drosha, and Dicer.

miRNAs are generated from precursor RNA Pol II transcripts that can either be intergenic or intragenic in origin [179,180]. Intergenic miRNAs have their own promoters and are transcribed into pri-miRNA precursors that are capped, and polyadenylated. More than 50% of miRNAs can be transcribed from intergenic regions as multicistronic transcripts [181]. Intragenic miRNAs are predominantly transcribed from introns of a host gene and released during splicing of the host gene. The pri-miRNAs have stem-loop structures that harbor the mature miRNA. The mature miRNA is generated by endonucleolytic cleavage by the nuclear Drosha/DGCR8 heterodimer to release a ~70 nt pre-miRNA hairpin with a 2 nt 3’ overhang. Exportin-5 (XPO5) and Ran-GTP facilitate nuclear export of the pre-miRNA hairpin by recognizing the 3’ overhang. In the cytoplasm, the pre-miRNA hairpin is cleaved by the RNAse III enzyme Dicer to generate the mature ~22 nt miRNA duplex. These mature miRNA duplexes associate with Ago proteins to form the RNA-induced silencing complex (RISC). Assembly of RISC requires degradation of one of the miRNA strands (passenger strand) while retaining the guide strand that ultimately base pairs with the mRNA 3’ UTR. The loading of the Ago-miRNA complex onto the mRNA recruits the mRNA degradation machinery that then targets the mRNA for either translational repression or decay (Figure 4A). 

The miRNA expression profile or “signatures” are predictors of cancer prognosis and are cancer hallmarks [182,183]. miRNA dependent pathways can be regulated at multiple levels upon DNA damage [181,184]. Similar to lncRNAs, the transcription of several miRNA genes, such as the miR-34 family in under the control of p53 and is altered upon DNA-damage [185]. Several miRNAs have been identified that directly regulate the abundance of DSB repair proteins at the posttranscriptional level (Table 3). The up- or down-regulation of miRNAs can affect protein stability of sensors of DNA damage such as *γ*H2AX [186,187], decreasing *γ*H2AX containing damage foci. The kinase ATM, a key regulator of the DNA damage response, is also under the control of several miRNAs (Table 3). Upregulation of miR-18a decreases the DNA damage response due to decreased ATM levels [188] whereas increased levels of miR-421, miR-100 and miR-101 leads to increased radiosensitivity [188,189,190]. The expression of DNA repair effectors is also under microRNA regulation (Table 3). In particular, the tumor suppressor BRCA1 and ssDNA binding protein RAD51, which are important for strand invasion in HR are tightly regulated by a number of miRNAs. MicroRNAs also control protein levels of cell cycle checkpoint factors and apoptosis regulators as has been reviewed [181,184]. Intriguingly, miRNAs can be secreted into body fluids such plasma and urine via exosomes and may be important mediators of DNA damage from radiation-targeted cells to abscopal normal cells, leading to the bystander effect [191]. Exosomes have been reported to contain as many as 764 miRNAs [192,193]. The miR-1246 is an example of a radiation-induced miRNA that is packaged into exosomes and is delivered to non-irradiated cells to decrease NHEJ efficiency by targeting the 3’ UTR of LIG4 [194]. These diverse roles of miRNAs in the DNA damage response and cancer have made them attractive targets for cancer therapy. 

### 4.2. Drosha- and Dicer-Dependent Small RNAs (DDRNAs)

Recent studies have shown that small ~21 nt RNAs are induced from the sequences around the site of damage in response to ionizing radiation. These RNAs are called diRNAs in *Arabidopsis thaliana* [71], qiRNAs in *Neurospora crassa* [215], endo-siRNAs in *Drosophila melanogaster* [216], and DDRNAs in human cells [217,218]. These RNAs are generated in a Dicer-dependent manner in all organisms and are distinct from miRNAs (Figure 4B). In mammals, down-regulation or inactivation of Drosha and Dicer impairs formation of DNA damage foci containing phosphor-ATM, MDC1, and 53BP1 in response to ionizing radiation [217,218]. Knockdown of Dicer and Drosha also results in a loss of the G1/S and G2/M checkpoints. Formation of DNA damage foci is RNA-dependent and removal of RNaseA and inhibiting transcription reduces foci formation. Using a chromosomally integrated reporter system and deep sequencing, recent studies show that foci formation requires small RNAs (20–35 nt) called DDRNAs that are generated by Drosha and Dicer from precursor RNAs at the damage site. The precise mechanism by which DDRNAs act is unclear, and it has been proposed that they act later in the DNA damage response and may function to recruit factors such as MDC1 and 53BP1 during HR [217]. A more recent study [219] shows that Drosha has an early role in DSB repair and is required for both HR and NHEJ. Drosha facilitates formation of RNA-DNA hybrids before resection can occur, and may interact directly with BRCA1. However, no DDRNAs were observed in this study [219] that examined endogenous break loci. However, all studies are in agreement for the role of RNA at DNA damage breaks and the requirement for miRNA processing enzymes such as Dicer and Drosha for efficient repair. 

## 5. Concluding Remarks

Small RNA and lncRNAs play intricate roles in the DNA damage response, although the mechanisms by which they act remain unclear. Understanding their modes of regulation in DDR provides new opportunities in cancer therapy. lncRNAs can act as guides, scaffolds, decoys in DNA repair. They may also be important for spatial regulation of DNA repair complexes by formation of DNA repair foci. Use of the latest deep sequencing technologies, imaging tools, combined with molecular and structural information on RNA and RNA-protein complexes is needed to understand non-coding RNA function in in DNA repair. 

## Figures and Tables

**Table 1:** LncRNAs involved in homologous recombination (HR) or non-homologous end joining (NHEJ).

lncRNA	Mechanism	DSB Repair Pathway	References
**1. p53 -linked lncRNAs**			
*(lincRNA)-p21*	Recruits hnRNPK to repress p21 transcription	HR	[101]
*PINT*	Interacts with PRC2 to silence transcription		[102,103]
*PANDA*	Negatively regulates apoptosis by sequestering the transcription factor NF-YA from pro-apoptotic gene site	HR	[104]
*DINO*	Interacts directly with p53 to stabilize it, inducing p53 target genes	HR	[105]
*LINP1*	May interact with Ku80/70 and DNAPKcs	NHEJ	[106]
*WRAP53*	Antisense lncRNA to p53 that regulates p53 levels	HR, NHEJ	[107]
*APELA*	Binds hnRNPL to block interaction with p53	HR	[104]
*MEG3*	Increases p53 levels	HR	
*LincROR*	Inhibits p53 translation after DNA damage	HR, NHEJ	[108,109]
*MALAT1*	Directly binds PARP1 and LIG3 to promote DNA repair; may promote p53 deacetylation via SIRT1 impairing its function	HR, NHEJ, Alt-NHEJ	[110,111]
*TUG-1*	Induced by p53 and binds PRC2 to repress cell-cycle genes	HR	[112,113]
*loc285194*	P53 target, tumor suppressor, down regulates miR-211	HR	[114]
**2. p53 independent lncRNAs**			
*DDSR1*	Sequesters the BRCA1-Rap80 complex via direct interactions with BRCA1	HR	[115]
*PCAT-1*	Represses BRCA2 expression in prostate cancer cells	HR	[116,117,118]
*lncRNA JADE*	Induced by ATM activation. Increases transcription of Jade1, a component of the HBO1 histone acetylation complex. Promotes H4 acetylation at K5, K8, K12	HR	[119]
*ANRIL*	Induced by ATM-mediated E2F1 activation. Regulates cell cycle checkpoints and apoptosis	HR	[120]
*BARD1 9´L*	Increases expression of a subset of BARD1 isoforms by sequestering miRNAs that normally destabilize BARD1 mRNAs	HR	[121]
*TERRA*	Interacts with Ku70/Ku80; facilitates Exo1 mediated DNA resection; promotes interaction of Mre11 with LSD1	HR, NHEJ	[122,123]
*TODRA*	Increases Rad51 transcription	HR	[124]
*MDC1-AS*	Upregulates the expression of the chromatin adaptor MDC1	HR	[125]
*Evf2*	Directly binds BRG1 and inhibits its ATPase dependent chromatin remodeling activity, may prevent Rad51 loading onto ssDNA via BRG1	HR	[126,127]
*CUPID1 and CUPID2*	Involved in pathway choice in switching from NHEJ to HR; promotes Rad51 recruitment to DSBs	HR	[128]

**Table 2:** Relevance of lncRNAs to development, disease, and inborn errors of metabolism.

lncRNA	Role is Disease and Development	References
**1. p53 -linked lncRNAs**		
*(lincRNA)-p21*	Type 2 diabetes, multiple cancer types	[151,152]
*PINT*	Breast cancer, pancreatic cancer, neuronal development, acute myocardial infarction	[153,154,155]
*PANDA*	Pancreatic cancer, Type 2 diabetes, osteosarcoma	[151,156,157]
*DINO*	multiple sclerosis	[158]
*LINP1*	Triple negative breast cancer, cervical cancer, prostate cancer	[106,139,140,141]
*WRAP53*	Unknown	
*APELA*	Ovarian cancer	[159]
*MEG3*	Huntington’s disease, gliomas	
*LincROR*	Non-small-cell lung cancer	[160]
*MALAT1*	multiple sclerosis	[158]
*TUG-1*	multiple sclerosis	[158]
*loc285194*	Osteosarcoma, ischemic heart failure	[161,162,163]
**2. p53 independent lncRNAs**		
*DDSR1*	Unknown	
*PCAT-1*	Multiple cancers including prostate cancer, bladder cancer, gastric cancer	[116,117,118,164,165]
*lncRNA JADE*	Unknown	
*ANRIL*	Coronary artery disease, COPD, multiple cancer types Type 2 diabetes, multiple sclerosis	[151,156,166,167]
*BARD1 9´L*	Multiple cancer types	[168]
*TERRA*	Alternative lengthening of telomeres via homology directed repair (ALT) cancers	[169]
*TODRA*	Epithelial ovarian cancer	[124]
*MDC1-AS*	Bladder cancer, gliomas, gastric cancer	[125,170,171]
*Evf2*	Important for embryonic neuronal development in mice	[172,173]
*CUPID1 and CUPID2*	Breast cancer	[128]

**Table 3:** miRNAs that directly target genes involved in homologous recombination (HR) or non-homologous end joining (NHEJ).

miRNAs	Target Gene	DSB Repair Pathway	References
miR-27a, miR-421, miR-101, miR-100, miR-18a, miR-181	*ATM*	HR	[188,189,190,195,196]
miR-101	*DNA-PKcs*	NHEJ	[190]
miR-124, miR-622	*Ku70*	NHEJ	[197,198]
miR-623, miR-526b, miR-622	*Ku80*	NHEJ	[198,199,200]
miR-1246	*LIG4*	NHEJ	[194]
miR-138, miR-24	*γ* *H2AX*	HR, NHEJ	[186,187]
miR-182-5p, miR-146a, miR-146b-5p, mir-1255b, miR-148b, miR-193b, miR-99, miR-28, let-7	*BRCA1*	HR	[188,201,202,203,204,205]
miR-19a, miR-19b, miR-1255b, miR-148b, miR-193b, let-7	*BRCA2*	HR	[204,205,206]
miR-96, miR-193a-3p, miR-506, miR-155, miR-1255b, miR-148b, miR-193b, miR-222, miR-107	*RAD51*	HR	[204,207,208,209,210,211,212]
let-7	*FANCD2*	HR	[205]
miR-210	*RAD52*	HR	[213]
miR-335	*CTIP*	HR	[214]
